# Quantum Coherences and Classical Inhomogeneities as Equivalent Thermodynamics Resources

**DOI:** 10.3390/e24040474

**Published:** 2022-03-29

**Authors:** Andrew Smith, Kanupriya Sinha, Christopher Jarzynski

**Affiliations:** 1 Department of Physics, University of Maryland, College Park, MD 20742, USA; andrew.maven.smith@gmail.com; 2 Department of Electrical and Computer Engineering, Princeton University, Princeton, NJ 08544, USA; kanu.sinha@asu.edu; 3 School of Electrical, Computer and Energy Engineering, Arizona State University, Phoenix, AZ 85287, USA; 4 Department of Chemistry and Biochemistry, University of Maryland, College Park, MD 20742, USA; 5 Institute for Physical Science and Technology, University of Maryland, College Park, MD 20742, USA

**Keywords:** quantum thermodynamics, quantum coherence, work extraction

## Abstract

Quantum energy coherences represent a thermodynamic resource, which can be exploited to extract energy from a thermal reservoir and deliver that energy as work. We argue that there exists a closely analogous classical thermodynamic resource, namely, energy-shell inhomogeneities in the phase space distribution of a system’s initial state. We compare the amount of work that can be obtained from quantum coherences with the amount that can be obtained from classical inhomogeneities, and find them to be equal in the semiclassical limit. We thus conclude that coherences do not provide a unique thermodynamic advantage of quantum systems over classical systems, in situations where a well-defined semiclassical correspondence exists.

## 1. Introduction

This paper considers the question: How much work W is extracted when a quantum system *S* undergoes a cyclic thermodynamic process? The answer depends on details such as the duration of the process; whether or not the system exchanges energy with heat baths along the way; how the system is driven during the process; and the system’s initial state, ${\hat{\rho }}_{i}$. We are specifically interested in the potential thermodynamic consequences of *energy coherences*—non-zero matrix elements $\langle m|{\hat{\rho }}_{i}|n\rangle$ for eigenstates of different energies—in the initial state. The thermodynamic utility of such coherences has been investigated in recent years [1,2,3,4,5,6,7,8,9,10,11,12,13,14,15,16,17,18,19], using a variety of approaches. Of particular relevance to the present paper, Kammerlander and Anders [9], using the definition of work [20,21] that we will use, have argued that if ${\hat{\rho }}_{i}$ contains coherences in the system’s energy basis, then more work can be extracted than would be possible in the absence of coherences. In this sense, quantum energy coherences represent a thermodynamic resource.

It seems natural to view the presence of energy coherences in ${\hat{\rho }}_{i}$ as a uniquely *quantum* thermodynamic resource, with no classical counterpart—in much the same way that superpositions of qubit states represent a quantum computational resource unavailable to classical computers [22]. We will argue otherwise. We will identify a classical analogue of quantum energy coherences, namely energy-shell *inhomogeneities* in the initial classical phase space distribution $\rho_{i}\left(\Gamma \right)$. We will show that the presence of such inhomogeneities in $\rho_{i}\left(\Gamma \right)$ allows more work to be extracted than would be possible in their absence. Thus, both quantum energy coherences and classical energy-shell inhomogeneities can be viewed as thermodynamic resources from which work can be extracted. We will further argue that for systems that support a well-defined semiclassical limit, a fair comparison reveals that equal amounts of work can be extracted from the two resources. We therefore conclude that quantum energy coherences do not provide a quantum “thermodynamic advantage”, as the same gain can be obtained from classical energy-shell inhomogeneities.

In Section 2, we introduce the framework and notation we will use to study a quantum system undergoing a cyclic thermodynamic process, in the presence of a thermal reservoir, and we analyze the work that can be extracted from energy coherences during such a process. In Section 3, we introduce the analogous classical framework and analyze the work that can be extracted from energy-shell inhomogeneities. In Section 4, we argue that when a fair comparison is made, the maximum amount of work that can be extracted in the quantum case is the same as that in the classical case. In Section 5, we extend these results to a broader class of processes. We conclude with a brief discussion in Section 6.

Throughout this paper, we will adopt an ensemble perspective, in which the state of an open quantum system is specified by a density matrix $\hat{\rho }$, and the state of a classical system is specified by a phase space distribution ρ(Γ) rather than a phase point Γ.

## 2. Quantum Setup and Notation

**Figure 1:**
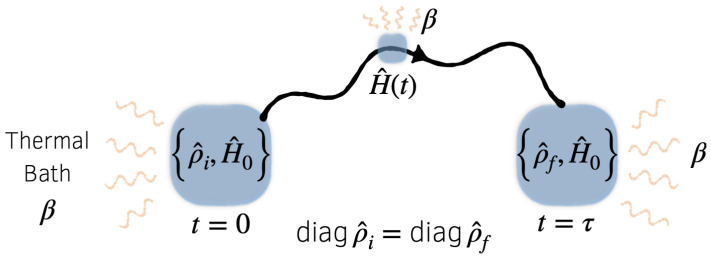
Schematic illustration of the quantum process described in the text. The system begins in state ${\hat{\rho }}_{i}$, then evolves in contact with a thermal bath to a final state ${\hat{\rho }}_{f}$ as the Hamiltonian is driven through a cycle from $\hat{H}\left(0\right)={\hat{H}}_{0}$ to $\hat{H}\left(\tau \right)={\hat{H}}_{0}$. We impose the constraint $\mathrm{diag}\;{\hat{\rho }}_{i}=\mathrm{diag}\;{\hat{\rho }}_{f}$, which indicates that the initial and final energy distributions are identical, while the coherences may differ.

Let *S* denote a quantum system of interest, and $\hat{H}$ its Hamiltonian. We consider the following situation, illustrated schematically in Figure 1: *S* is prepared in an initial state ${\hat{\rho }}_{i}$ at time t=0, then from t=0 to τ it evolves in time as its Hamiltonian is varied according to a schedule, or *protocol*, $\hat{H}\left(t\right)$. We take this process to be cyclic, in the sense that

(1) $$\hat{H}\left(0\right)=\hat{H}\left(\tau \right)={\hat{H}}_{0}$$

where ${\hat{H}}_{0}$ is a fixed *reference Hamiltonian*. We then ask the question: How much work is extracted during this cyclic process?

We assume the reference Hamiltonian ${\hat{H}}_{0}$ has a discrete, non-degenerate spectrum with eigenstates $|n\rangle$ and eigenvalues $\epsilon_{n}$. The assumption of non-degeneracy ensures an unambiguously defined energy basis in which coherence can be considered. It further implies that no operators commute with ${\hat{H}}_{0}$, aside from ones that are functions of ${\hat{H}}_{0}$ itself:

(2) $$\left[\hat{K},{\hat{H}}_{0}\right]=0\;\;\;\mathrm{iff}\;\;\;\hat{K}=k\left({\hat{H}}_{0}\right)$$

for some scalar function k(·) of a single variable.

During the cyclic process described above, the system is in contact with a thermal bath *B*, at temperature $\beta^{-1}$. As a result, the evolution of *S* is not unitary, rather, we will say that *S* evolves under *isothermal dynamics*. This terminology is not meant to suggest that the system’s temperature is constant, or even well-defined, merely that the system is in contact with a bath whose bulk temperature $\beta^{-1}$ is well-defined. We will not specify the equations of motion for the system, as our discussion will be relatively insensitive to the exact dynamics used to model the system’s evolution. However, we will demand that the isothermal dynamics of *S* satisfy the following thermodynamically motivated conditions: (1) if $\hat{H}$ is held fixed then the system relaxes to the canonical equilibrium state, and (2) the dynamics support a generalized second law linking suitably defined notions of free energy and work.

More precisely, condition (1) means that if $\hat{H}$ is fixed, then the isothermal dynamics cause the system to relax to the equilibrium state

(3) $$\hat{\pi }=\frac{1}{Z^{q}}e^{-\beta \hat{H}}$$

where

(4) $$Z^{q}\left(\hat{H}\right)=\mathrm{Tr}\;e^{-\beta \hat{H}}\;,\;F^{q,\mathrm{eq}}\left(\hat{H}\right)=-\beta^{-1}\ln Z^{q}\left(\hat{H}\right)$$

are the partition function and free energy associated with this state. (The superscript *q* stands for “quantum” and distinguishes this case from the classical setup that will be introduced later. The dependence of $Z^{q}$ and $F^{q,\mathrm{eq}}$ on β is notationally suppressed.) We assume this relaxation occurs over a finite characteristic timescale $\tau_{rel}$. As a consequence, if the system Hamiltonian is varied quasistatically, then the state of *S* tracks the instantaneous equilibrium state: $\hat{\rho }\left(t\right)=\hat{\pi }\left(t\right)$, where $\hat{\pi }\left(t\right)$ is the canonical state associated with $\hat{H}\left(t\right)$. In this quasistatic limit, the system’s evolution is isothermal in the strong sense of the word: its temperature is well-defined and constant at all times. A system that evolves under a detailed balanced Lindblad master equation satisfies condition (1) [23].

By condition (2), we mean that the system obeys a generalized second law

(5) $$W^{q}\leq -\Delta F^{q}=F^{q}\left(0\right)-F^{q}\left(\tau \right)$$

where the work *extracted*, non-equilibrium free energy, internal energy, and entropy are respectively defined by the following functional and functions of $\hat{\rho }\left(t\right)$ and $\hat{H}\left(t\right)$:

(6) $$\begin{array}{ccc} W^{q}\left[\hat{\rho }\left(t\right),\hat{H}\left(t\right)\right] & = & -\int_{0}^{\tau }\mathrm{Tr}\left[\frac{d\hat{H}}{dt}\hat{\rho }\right]dt \end{array}$$

(7) $$\begin{array}{ccc} F^{q}\left(\hat{\rho },\hat{H}\right) & = & \;U^{q}-S^{q}/\beta \end{array}$$

(8) $$\begin{array}{ccc} U^{q}\left(\hat{\rho },\hat{H}\right) & = & \;\mathrm{Tr}[\hat{H}\hat{\rho }] \end{array}$$

(9) $$\begin{array}{ccc} S^{q}\left(\hat{\rho }\right) & = & -\mathrm{Tr}[\hat{\rho }\;\ln\hat{\rho }]\geq 0. \end{array}$$

For convenience, as in Equation (5), we will often use the shorthand $X\left(t\right)\equiv X(\hat{\rho }\left(t\right),\hat{H}\left(t\right))$, or the even more concise $X_{i}=X\left(0\right)$ and $X_{f}=X\left(\tau \right)$, where X stands for $F^{q}$, $U^{q}$, or $S^{q}$, or the classical counterparts of these quantities, defined below in Section 3.

Equations (7)–(9) generalize familiar equilibrium notions [24] of free energy, internal energy, and entropy to non-equilibrium states $\hat{\rho }$ [25]. They reduce to the usual equilibrium values when $\hat{\rho }=\hat{\pi }$. The bound given by Equation (5) is not restricted to transitions between equilibrium states, and has been derived using a variety of approaches for modeling the dynamics of a quantum system in contact with a thermal reservoir, see, e.g., Refs. [26,27,28,29,30]. Note that we follow engineering convention and work extraction is positive. While Equation (6) should be interpreted as the *average* work extracted from an ensemble, fluctuations will not be considered in this paper, hence we will simply refer to Equation (6) as extracted work.

The non-equilibrium free energy defined by Equation (7) can equivalently be written as

(10) $$F^{q}\left(\hat{\rho },\hat{H}\right)=F^{q,\mathrm{eq}}\left(\hat{H}\right)+\beta^{-1}D\left(\hat{\rho }|\hat{\pi }\right)$$

where $\hat{\pi }$ and $F^{q,\mathrm{eq}}$ are given by Equations (3) and (4), and

(11) $$D\left({\hat{\rho }}_{1}|{\hat{\rho }}_{2}\right)=\mathrm{Tr}\left[{\hat{\rho }}_{1}\left(\ln{\hat{\rho }}_{1}-\ln{\hat{\rho }}_{2}\right)\right]\geq 0$$

is the quantum relative entropy, or Kullback–Leibler divergence [31], between arbitrary states ${\hat{\rho }}_{1}$ and ${\hat{\rho }}_{2}$. For a cyclic process, as defined above, Equation (5) becomes

(12) $$W^{q}\leq \beta^{-1}\left[D\left({\hat{\rho }}_{i}|{\hat{\pi }}_{0}\right)-D\left({\hat{\rho }}_{f}|{\hat{\pi }}_{0}\right)\right]$$

where ${\hat{\rho }}_{i,f}$ are the states of the system at t=0,τ, and ${\hat{\pi }}_{0}$ is the equilibrium state associated with the reference Hamiltonian ${\hat{H}}_{0}$. Although relative entropy $D({\hat{\rho }}_{1}|{\hat{\rho }}_{2})$ is not a proper distance measure, it vanishes when ${\hat{\rho }}_{1}={\hat{\rho }}_{2}$ and is strictly positive otherwise, and can be viewed as quantifying the degree to which ${\hat{\rho }}_{1}$ differs from ${\hat{\rho }}_{2}$. In this sense, Equation (12) implies that the extracted work is bounded from above by the degree to which the system is brought closer to the equilibrium state ${\hat{\pi }}_{0}$, during the cyclic process. This interpretation is in agreement with the intuition, from classical thermodynamics, that non-equilibrium states represent a thermodynamic resource: work can be extracted by cleverly facilitating a system’s evolution toward equilibrium.

We take Equation (6) as our definition of work for several reasons. First, it is an established notion of thermodynamic work in quantum systems [20,21,32]. Moreover, it agrees with the notion of average work derived from the quantum work (quasi)distribution in Ref. [33], which satisfies a fluctuation theorem. Finally, this definition closely resembles those used in classical stochastic thermodynamics [34,35] and, as we will see in later sections, it allows us to establish connections with results from classical statistical physics. For the special case of isolated quantum systems, the definition given by Equation (6) is called “untouched work” in Ref. [36]. We will not discuss here how (or whether) Equation (6) connects to the traditional thermodynamic concept of raising a mass against gravity, or otherwise delivering energy to a work reservoir [24]; this question involves subtle issues related to backaction as well as potential quantum coherences in the work reservoir.

We note that other definitions of work are also commonly used in quantum thermodynamics, particularly when fluctuations in work are of interest. For instance, defining a work distribution according to the two-time energy measurement protocol [37,38,39] leads to a mean value that differs from Equation (6) whenever the initial state ${\hat{\rho }}_{i}$ has non-vanishing energy coherences. Additionally, some definitions of work developed in quantum resource theory [40] have a so-called work-locking property [41] which prevents the extraction of work from coherence. These resource theory definitions, which explicitly model the heat bath and demand that work be transferred deterministically, also differ from Equation (6).

### Removing Coherences

To this point, we have discussed subjecting the system *S* to a cyclic process under isothermal dynamics. Now, following Ref. [9], we impose an additional condition:

(13) $$\mathrm{diag}\;{\hat{\rho }}_{f}=\mathrm{diag}\;{\hat{\rho }}_{i}$$

where

(14) $$\mathrm{diag}\;\hat{\rho }=\sum_{n}|n\rangle \langle n|\hat{\rho }|n\rangle \langle n|$$

is the density matrix obtained from $\hat{\rho }$ by setting to zero its off-diagonal elements, in the reference energy basis. In other words, we now restrict ourselves to processes that alter the system’s energy coherences $\langle m|\hat{\rho }|n\rangle$, m≠n, while leaving the probabilities $\langle n|\hat{\rho }|n\rangle$ unchanged. We will refer to Equation (13), and to its classical counterpart, Equation (42), as the *isoenergetic constraint*. As in Ref. [9], our motivation for imposing this condition is to isolate and accentuate the thermodynamic implications of quantum energy coherences. From Equation (13), it follows that $\mathrm{Tr}\left[{\hat{H}}_{0}{\hat{\rho }}_{i}\right]=\mathrm{Tr}\left[{\hat{H}}_{0}{\hat{\rho }}_{f}\right]$, i.e.,

(15) $$U_{i}^{q}=U_{f}^{q}$$

which in turn implies that the generalized second law, Equation (5), becomes

(16) $$W^{q}\leq \beta^{-1}\left(S_{f}^{q}-S_{i}^{q}\right).$$

This bound relates the maximum extractable work to the change in the system’s entropy. The thermodynamic interpretation is clear: since the system’s energy undergoes no net change (Equation (15)), the only way to extract work is to withdraw energy from the bath, causing the entropy of the bath to decrease by an amount $\beta W^{q}$. This decrease in the bath’s entropy must be compensated, or over-compensated, by an increase in the entropy of the system, as reflected by Equation (16).

We are now in a position to investigate the maximum amount of work that can be extracted from energy coherences. For a given reference Hamiltonian ${\hat{H}}_{0}$ and initial state ${\hat{\rho }}_{i}$, let $W^{q🟉}$ denote the maximum extracted work, over all protocols $\hat{H}\left(t\right)$ that begin and end in ${\hat{H}}_{0}$, subject to the isoenergetic constraint (13). Since the right side of Equation (16) is a function of ${\hat{\rho }}_{i}$ and ${\hat{\rho }}_{f}$, we can place a bound on $W^{q🟉}$ by maximizing that function with respect to ${\hat{\rho }}_{f}$:

(17) $$W^{q🟉}\leq \beta^{-1}\max_{{\hat{\rho }}_{f}|\mathrm{diag}\;{\hat{\rho }}_{f}=\mathrm{diag}\;{\hat{\rho }}_{i}}\left[S^{q}\left({\hat{\rho }}_{f}\right)-S^{q}\left({\hat{\rho }}_{i}\right)\right]$$

For fixed diagonal elements of a density matrix $\hat{\rho }$, the value of $S^{q}=-\mathrm{Tr}\hat{\rho }\ln\hat{\rho }$ is maximized when the off-diagonal elements are all zero. We therefore obtain

(18) $$W^{q🟉}\leq \beta^{-1}\left[S^{q}\left(\mathrm{diag}\;{\hat{\rho }}_{i}\right)-S^{q}\left({\hat{\rho }}_{i}\right)\right].$$

This result does not yet tell us whether the bound can be saturated, that is, whether there exist protocols for extracting this amount of work. Rather, it states that under no circumstances can we extract more than this much work, in a cyclic, isothermal process satisfying Equation (13). Moreover, if a protocol for saturating this bound exists, then that protocol will result in the system ending in the state $\mathrm{diag}\;{\hat{\rho }}_{i}$ at t=τ. In other words, the saturating protocol (if it exists) removes all energy coherences from the system’s initial state, and effectively converts these coherences into extracted work.

In fact, protocols for saturating the bound given by Equation (18) do exist [9,28]. A simple example is given by:

(19) $$\hat{H}\left(t\right)=\left\{\begin{array}{cc} {\hat{H}}_{0} & \;t\leq 0\\ -\beta^{-1}\ln\left[\left(1-\lambda \right){\hat{\rho }}_{i}+\lambda \left(\mathrm{diag}\;{\hat{\rho }}_{i}\right)\right] & \;0<t<\tau \\ {\hat{H}}_{0} & \;\tau \leq t \end{array}\right.$$

where λ≡t/τ varies from 0 to 1 during the process, and τ is taken to be sufficiently large that the process is quasistatic. This protocol can be understood as follows. At the start of the process, there is a sudden change, or *quench*, in the system’s Hamiltonian, from ${\hat{H}}_{0}$ at t=0 to $-\beta^{-1}\ln{\hat{\rho }}_{i}$ at $t=0^{+}$. Thus, at $t=0^{+}$ the system’s state ${\hat{\rho }}_{i}$ is in equilibrium with respect to the immediate post-quench Hamiltonian. (The term “quench” is often used in situations in which the system is in equilibrium before the quench, and out of equilibrium after it. Thus, the first step of this protocol (19) might be viewed as an *anti*-quench.) From $t=0^{+}$ to $\tau^{-}$, the Hamiltonian is varied quasistatically from $-\beta^{-1}\ln{\hat{\rho }}_{i}$ to $-\beta^{-1}\ln\left(\mathrm{diag}\;{\hat{\rho }}_{i}\right)$, and the system is dragged through the corresponding sequences of equilibrium states, from ${\hat{\rho }}_{i}$ to $\mathrm{diag}\;{\hat{\rho }}_{i}$—see comments after Equation (4). At t=τ, a second quench abruptly returns the Hamiltonian to ${\hat{H}}_{0}$, completing the cycle. The evolution of the system’s state is thus given by

(20) $$\hat{\rho }\left(t\right)=\left\{\begin{array}{cc} {\hat{\rho }}_{i} & \;t=0\\ \left(1-\lambda \right){\hat{\rho }}_{i}+\lambda \left(\mathrm{diag}\;{\hat{\rho }}_{i}\right) & \;0<t<\tau \\ \mathrm{diag}\;{\hat{\rho }}_{i} & \;\tau \leq t. \end{array}\right.$$

We show in Appendix A that the work extracted during this process is given by the right side of Equation (18), that is the bound is saturated. Hence, under the isoenergetic constraint (13), work extraction is optimized by removing all coherences from the system’s state, and the value of this optimized work is:

(21) $$W^{q🟉}=\beta^{-1}\left[S^{q}\left(\mathrm{diag}\;{\hat{\rho }}_{i}\right)-S^{q}\left({\hat{\rho }}_{i}\right)\right].$$

This result is equivalent to Equation (1) of Kammerlander and Anders [9].

## 3. Classical Setup and Notation

Now, imagine a classical system with *N* degrees of freedom and phase space variables

(22) $$\Gamma =(x_{1},...,x_{N},p_{1},...,p_{N}).$$

**Figure 2:**
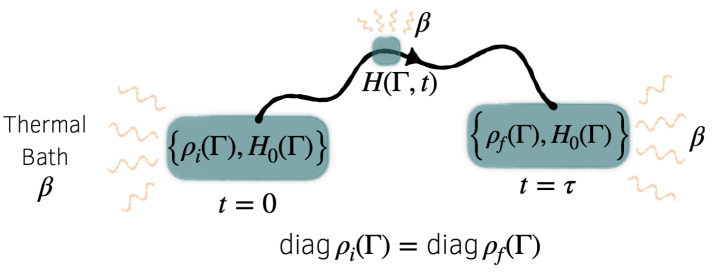
Schematic illustration of the classical process. The system begins in state $\rho_{i}\left(\Gamma \right)$, then evolves in contact with a thermal bath to a final state $\rho_{f}\left(\Gamma \right)$ as the Hamiltonian is driven through a cycle from $H\left(\Gamma ,0\right)=H_{0}\left(\Gamma \right)$ to $H\left(\Gamma ,\tau \right)=H_{0}\left(\Gamma \right)$. The constraint $\mathrm{diag}\;\rho_{i}\left(\Gamma \right)=\mathrm{diag}\;\rho_{f}\left(\Gamma \right)$ indicates that the initial and final energy distributions are identical, while inhomogeneities may differ.

Adopting (as in the quantum case) an ensemble perspective, let the system’s state at time *t* be described by a phase space density ρ(Γ,t). We will consider a thermodynamic process in which the system begins in a state $\rho \left(\Gamma ,0\right)=\rho_{i}\left(\Gamma \right)$, then evolves from t=0 to τ as its Hamiltonian is varied according to a cyclic protocol H(Γ,t), with

(23) $$H\left(\Gamma ,0\right)=H\left(\Gamma ,\tau \right)=H_{0}\left(\Gamma \right)$$

where $H_{0}\left(\Gamma \right)$ specifies a reference Hamiltonian; see Figure 2. We assume that no observables commute with $H_{0}\left(\Gamma \right)$ under the Poisson bracket, except those that are functions of $H_{0}$:

(24) $$\left\{K,H_{0}\right\}=0\;\;\;\mathrm{iff}\;\;\;K\left(\Gamma \right)=k\left(H_{0}\left(\Gamma \right)\right)$$

for some function k(·) (compare with Equation (2)). This assumption implies that energy is the only non-trivially conserved quantity along all trajectories Γ(t) obeying Hamiltonian dynamics $d\Gamma /dt=\{\Gamma ,H_{0}\}$. (This conclusion follows from the identity $\left(d/dt\right)A\left(\Gamma \left(t\right)\right)=\left\{A,H_{0}\right\}$, which applies to any observable A(Γ) and any trajectory Γ(t) obeying $d\Gamma /dt=\{\Gamma ,H_{0}\}$). This is a necessary but not sufficient condition for the dynamics to be *ergodic* on constant-energy surfaces in phase space—an assumption often made in statistical physics. (Roughly speaking, ergodicity means that a generic Hamiltonian trajectory of energy *E* visits all regions of the surface $H_{0}=E$, given sufficient time.) For our purposes, we do not need the assumption of ergodicity, only the weaker assumption given by Equation (24).

If the system were thermally isolated, then its state ρ(Γ,t) would evolve under the Liouville equation, ∂ρ/∂t={H,ρ}. However, we assume that the system is in contact with a thermal bath as it undergoes the cyclic process, hence its evolution follows classical isothermal dynamics, rather than Hamiltonian dynamics. As in the quantum case, we will not specify the equations of motion that describe the isothermal dynamics, but we will make the following assumptions.

(1) If the system’s Hamiltonian is held fixed, then the isothermal dynamics drive the system to the equilibrium state

(25) $$\pi \left(\Gamma \right)=\frac{1}{Z^{c}}e^{-\beta H(\Gamma )}$$

with partition function and free energy

(26) $$Z^{c}\left[H\left(\Gamma \right)\right]=\int d\Gamma \;e^{-\beta H(\Gamma )}\;,\;F^{c,\mathrm{eq}}\left[H\left(\Gamma \right)\right]=-\beta^{-1}\ln\left(Z^{c}/h^{N}\right).$$

Here, *h* is a constant with dimensions of action that ensures the argument of the logarithm is dimensionless. We choose *h* to coincide with Planck’s constant as this will facilitate comparisons of quantum and classical work extraction in Section 4. We assume this relaxation takes place over a finite timescale $\tau_{\mathrm{rel}}$. As a consequence, if H(Γ,t) is varied quasistatically, then the system’s state follows the instantaneous equilibrium state, ρ(Γ,t)=π(Γ,t).

(2) When the system evolves over a time interval 0≤t≤τ under isothermal dynamics and a time-dependent Hamiltonian H(Γ,t), it obeys a generalized second law

(27) $$W^{c}\leq -\Delta F^{c}$$

with

(28) $$\begin{array}{cc} W^{c}\left[\rho \left(\Gamma ,t\right),H\left(\Gamma ,t\right)\right]= & -\int_{0}^{\tau }\left(\int \frac{\partial H}{\partial t}\rho d\Gamma \right)dt \end{array}$$

(29) $$\begin{array}{cc} F^{c}\left[\rho \left(\Gamma \right),H\left(\Gamma \right)\right]= & \;\;U^{c}-S^{c}/\beta \end{array}$$

(30) $$\begin{array}{cc} U^{c}\left[\rho \left(\Gamma \right),H\left(\Gamma \right)\right]= & \int H(\Gamma )\rho (\Gamma )d\Gamma \end{array}$$

(31) $$\begin{array}{cc} S^{c}\left[\rho \left(\Gamma \right)\right]= & -\int \rho \left(\Gamma \right)\ln[h^{N}\rho \left(\Gamma \right)]d\Gamma . \end{array}$$

Unlike the quantum von Neumann entropy (9) which is always non-negative, the classical Shannon differential (or continuous) entropy (31) can become arbitrarily negative for probability distributions that are highly concentrated in phase space, as we will see in Section 4.1. (For brevity, we will henceforth refer to the Shannon differential entropy simply as the Shannon entropy.) As with the quantum bound (Equation (5)), Equation (27) is not restricted to transitions between equilibrium states, and has been derived under a variety of modeling approaches, see, e.g., Refs. [26,27,28,29,30,42,43,44].

The classical non-equilibrium free energy (29) can be rewritten as

(32) $$F^{c}\left[\rho ,H\right]=F^{c,\mathrm{eq}}\left[H\right]+\beta^{-1}D\left[\rho |\pi \right]$$

where

(33) $$D\left[\rho_{1}|\rho_{2}\right]=\int d\Gamma \;\rho_{1}\left(\Gamma \right)\ln\frac{\rho_{1}\left(\Gamma \right)}{\rho_{2}\left(\Gamma \right)}\geq 0$$

is the classical relative entropy or Kullback–Leibler divergence. Thus, for a cyclic process, Equation (27) becomes

(34) $$W^{c}\leq \beta^{-1}(D\left[\rho_{i}|\pi_{0}\right]-D\left[\rho_{f}|\pi_{0}\right])$$

where $\rho_{i}\left(\Gamma \right)=\rho \left(\Gamma ,0\right)$ and $\rho_{f}\left(\Gamma \right)=\rho \left(\Gamma ,\tau \right)$ are the system’s initial and final states, and $\pi_{0}\left(\Gamma \right)$ is the equilibrium state for the reference Hamiltonian. As in the quantum case (Equation (12)), the right side of Equation (34) provides a measure of the degree to which the process brings the system closer to equilibrium.

### 3.1. Energy-Shell Inhomogeneities

The evident similarity between the quantum framework for cyclic isothermal processes described by Equations (1)–(12) and the classical framework of Equations (23)–(34) motivates us to seek a classical analogue of the statement that quantum energy coherences represent a thermodynamic resource. As a step in this direction, we note that in the quantum case, density matrices that are stationary under the unitary evolution generated by ${\hat{H}}_{0}$ are exactly those that lack energy coherences in the eigenbasis of ${\hat{H}}_{0}$:

(35) $$\frac{d\hat{\rho }}{dt}=\frac{1}{i\hbar }\left[{\hat{H}}_{0},\hat{\rho }\right]=0\;\mathrm{iff}\;\hat{\rho }=\mathrm{diag}\;\hat{\rho }.$$

In the classical case, phase space densities that are stationary under the Hamiltonian dynamics generated by $H_{0}\left(\Gamma \right)$ are exactly those that are functions of $H_{0}\left(\Gamma \right)$:

(36) $$\frac{\partial \rho }{\partial t}=\left\{H_{0},\rho \right\}=0\;\mathrm{iff}\;\rho \left(\Gamma \right)=k\left(H_{0}\left(x\right)\right)$$

for some function k(·). (Equations (2) and (24) are needed for the “only if” parts of Equations (35) and (36).) These observations suggest that we ought to view phase space distributions of the form $\rho =k(H_{0})$ as analogues of density matrices that are diagonal in the eigenbasis of ${\hat{H}}_{0}$.

To pursue this idea, let η(E) denote the distribution of energies associated with a phase space density ρ(Γ):

(37) $$\eta \left(E\right)=\int d\Gamma \;\rho \left(\Gamma \right)\;\delta [E-H_{0}\left(\Gamma \right)].$$

In addition, let $\omega_{E}\left(\Gamma \right)$ denote the classical microcanonical density of energy *E*:

(38) $$\omega_{E}\left(\Gamma \right)=\lim_{\Delta E\to 0}\frac{I_{\left[E,E+\Delta E\right]}\left(H_{0}\left(\Gamma \right)\right)}{\int d\Gamma^{\prime }\;I_{\left[E,E+\Delta E\right]}\left(H_{0}\left(\Gamma^{\prime }\right)\right)}=\frac{\delta [E-H_{0}\left(\Gamma \right)]}{\Omega (E)}$$

where $I_{\left[E,E+\Delta E\right]}\left(\cdot \right)$ is the indicator function over the interval [E,E+ΔE] (that is, $I_{\left[a,b\right]}\left(x\right)=1$ when x∈[a,b], otherwise $I_{\left[a,b\right]}\left(x\right)=0$), and

(39) $$\Omega \left(E\right)=\int d\Gamma \;\delta [E-H_{0}\left(\Gamma \right)]$$

is the classical density of states. The microcanonical density $\omega_{E}\left(\Gamma \right)$ is singular, uniformly distributed over the *energy shell E* (the level set $H_{0}=E$), and zero elsewhere. Here, “uniformly distributed” is defined by Equation (38): as ΔE approaches zero, the phase space density remains uniform, with respect to the Liouville measure $d^{N}x\;d^{N}p$, in the region between shells *E* and E+ΔE, and zero elsewhere.

Using Equations (37)–(39), a phase space density of the form $\rho =k(H_{0})$ can be written as

(40) $$\rho \left(\Gamma \right)=\int dE\;\eta \left(E\right)\;\omega_{E}\left(\Gamma \right)$$

with η(E)=k(E)Ω(E). Such a density is a statistical mixture of microcanonical ensembles (just as a diagonal density matrix is a mixture of energy eigenstates: $\hat{\rho }=\mathrm{diag}\;\hat{\rho }=\sum_{n}p_{n}|n\rangle \langle n|$), hence ρ(Γ) is uniform, or *homogeneous*, over any specific energy shell *E*, while its value differs from one shell to another. By contrast, a phase space density that is not of the form $\rho =k(H_{0})$ is *inhomogeneous* on energy shells: there exist points Γ and $\Gamma^{\prime }$ such that $H_{0}\left(\Gamma \right)=H_{0}\left(\Gamma^{\prime }\right)$ but $\rho \left(\Gamma \right)\neq \rho \left(\Gamma^{\prime }\right)$.

We will henceforth use the terms homogeneous/inhomogeneous to distinguish between phase space densities that can/cannot be written as $\rho =k(H_{0})$. For instance, the equilibrium distribution $\pi_{0}\left(\Gamma \right)\propto \exp-\beta H_{0}\left(\Gamma \right)$ is a homogeneous density. By the stationarity argument given above (Equations (35) and (36)), homogeneous phase space densities will be viewed as classical counterparts of diagonal density matrices, and inhomogeneous densities as counterparts of quantum states with energy coherences. In other words, for our purposes *the counterparts of quantum energy coherences are classical energy-shell inhomogeneities*.

We introduce the notation

(41) $$\mathrm{diag}\;\rho \left(\Gamma \right)=\int dE\;\eta \left(E\right)\;\omega_{E}\left(\Gamma \right)$$

with η(E) given by Equation (37), to denote the phase space density obtained by “homogenizing” ρ(Γ). That is, diagρ is the homogeneous density that has the same energy distribution as ρ.

### 3.2. Removing Inhomogeneities

Let us now focus our attention on classical, cyclic isothermal processes that satisfy the isoenergetic constraint (compare with Equation (13)):

(42) $$\mathrm{diag}\;\rho_{f}\left(\Gamma \right)=\mathrm{diag}\;\rho_{i}\left(\Gamma \right)$$

where $\rho_{i,f}$ denote the system’s initial and final states. Such processes leave the energy distribution undisturbed, $\eta_{i}\left(E\right)=\eta_{f}\left(E\right)$, while allowing energy-shell inhomogeneities to change. Equation (42) implies

(43) $$U_{i}^{c}=U_{f}^{c}$$

hence, Equation (27) becomes

(44) $$W^{c}\leq \beta^{-1}\left(S_{f}^{c}-S_{i}^{c}\right).$$

Let $W^{c🟉}$ denote the maximum amount of work that can be extracted, over all conceivable cyclic protocols, for a given reference Hamiltonian $H_{0}\left(\Gamma \right)$ and initial state $\rho_{i}\left(\Gamma \right)$. Equation (44) implies

(45) $$\begin{array}{ccc} W^{c🟉} & \leq & \;\beta^{-1}\max_{\rho_{f}|\mathrm{diag}\;\rho_{f}=\mathrm{diag}\;\rho_{i}}\left(S^{c}\left[\rho_{f}\right]-S^{c}\left[\rho_{i}\right]\right)\\ & = & \beta^{-1}\left(S^{c}\left[\mathrm{diag}\;\rho_{i}\right]-S^{c}\left[\rho_{i}\right]\right) \end{array}$$

since, among all states with a given energy distribution, the Shannon entropy is maximized by the homogeneous state (This follows from the fact that Shannon entropy increases under coarse-graining, which in turn is a consequence of Jensen’s inequality, 〈lnx〉≤ln〈x〉).

Similarly to the quantum case (Equation (19)), the bound given by Equation (45) is saturated [27,28] by the protocol

(46) $$H\left(\Gamma ,t\right)=\left\{\begin{array}{cc} H_{0}\left(\Gamma \right) & \;t\leq 0\\ -\beta^{-1}\ln\left[\left(1-\lambda \right)\rho_{i}\left(\Gamma \right)+\lambda \;\mathrm{diag}\;\rho_{i}\left(\Gamma \right)\right] & \;0<t<\tau \\ H_{0}\left(\Gamma \right) & \;\tau \leq t \end{array}\right.$$

with λ=t/τ, and τ sufficiently long that the process is effectively quasistatic. The protocol begins with a classical quench at t=0. Immediately after this quench, the system’s state $\rho_{i}\left(\Gamma \right)$ is in equilibrium with its instantaneous Hamiltonian, $H\left(\Gamma ,0^{+}\right)=-\beta^{-1}\ln\rho_{i}\left(\Gamma \right)$. During the interval t∈(0,τ), the quasistatic switching of the Hamiltonian drags the system through a sequence of equilibrium states from $\rho_{i}$ to $\rho_{f}=\mathrm{diag}\;\rho_{i}$, and at t=τ the cyclic process is completed by suddenly returning the Hamiltonian to $H_{0}$. The evolution of the system’s state ρ(Γ,t) is entirely analogous to that given by Equation (20). Summing over the work extracted during the initial quench, the quasistatic driving, and the final quench, we find (see Appendix A) that the total extracted work is

(47) $$W^{c🟉}=\beta^{-1}\left(S^{c}\left[\mathrm{diag}\;\rho_{i}\right]-S^{c}\left[\rho_{i}\right]\right),$$

i.e., the bound in Equation (45) is saturated. By Equation (44), any protocol satisfying Equation (42) that would bring the system to a final state $\rho_{f}\neq \mathrm{diag}\;\rho_{i}$ would necessarily result in less work extracted.

## 4. Quantum–Classical Comparison

We have seen that the maximum work extracted in the quantum case, subject to the isoenergetic constraint, $\mathrm{diag}\;{\hat{\rho }}_{i}=\mathrm{diag}\;{\hat{\rho }}_{f}$, is achieved by quasistatically removing all energy coherences from the system’s initial state: ${\hat{\rho }}_{i}\to \mathrm{diag}\;{\hat{\rho }}_{i}$. Similarly, the maximum work extracted in the classical case is achieved by quasistatically removing all energy-shell inhomogeneities. The optimized work values $W^{q🟉}$ and $W^{c🟉}$ are given by Equations (21) and (47). The close similarity between these results supports our view that classical energy-shell inhomogeneities are thermodynamic counterparts of quantum energy coherences. Both are resources that can be leveraged to extract work.

While the expressions for $W^{q🟉}$ and $W^{c🟉}$ are nearly identical, it still remains to compare them quantitatively. Ideally, we would like to compare the values of $W^{q🟉}$ and $W^{c🟉}$ for a given quantum reference Hamiltonian ${\hat{H}}_{0}$ and initial state ${\hat{\rho }}_{i}$, and appropriately defined classical counterparts $H_{0}\left(\Gamma \right)$ and $\rho_{i}\left(\Gamma \right)$. To this end, throughout this section and the next we assume that ${\hat{H}}_{0}$ is a function of position and momentum operators $\left({\hat{x}}_{1},\cdots {\hat{x}}_{N}\right)$ and $\left({\hat{p}}_{1},\cdots {\hat{p}}_{N}\right)$, and we further assume that ${\hat{H}}_{0}$ has a well-defined counterpart $H_{0}\left(\Gamma \right)$. This condition is satisfied, for instance, by Hamiltonians of the kinetic-plus-potential form ${\hat{H}}_{0}=K\left({\hat{p}}_{1},\cdots {\hat{p}}_{N}\right)+V\left({\hat{x}}_{1},\cdots {\hat{x}}_{N}\right)$, for which $H_{0}\left(\Gamma \right)$ is obtained by replacing momentum and position operators with classical momentum and position variables.

Identifying a correspondence between quantum and classical states $\hat{\rho }$ and ρ(Γ) is trickier. Common approaches that map density operators into phase space distributions [45,46] suffer from undesirable properties. For instance, neither the Wigner [47] nor Husimi [48] function representation of the quantum thermal state corresponds to the classical thermal phase space distribution. Additionally, the Wigner function in general can become negative while the Husimi function depends on the choice of coherent states.

To circumvent such issues, we will compare quantum and classical *energy distributions* rather than individual states. Instead of focusing on the maximum work that can be extracted from a particular initial state, we will consider the maximum work that can be extracted given a particular initial energy distribution. We begin by defining *energy equivalence classes* in Section 4.1, then in Section 4.2 and Section 4.3 we compare maximum work values for corresponding quantum and classical energy equivalence classes.

### 4.1. Energy Equivalence Classes

We define a quantum energy equivalence class to consist of all states $\hat{\rho }$ that share a particular energy distribution, that is, a particular set of diagonal density matrix elements, with respect to ${\hat{H}}_{0}$. An example is the thermal energy equivalence class given by

(48) $$\Pi^{q}=\left\{\hat{\rho }\;|\mathrm{diag}\;\hat{\rho }={\hat{\pi }}_{0}\right\}$$

where ${\hat{\pi }}_{0}=\exp\left(-\beta {\hat{H}}_{0}\right)/Z^{q}$ is the thermal equilibrium state. In addition to the state ${\hat{\pi }}_{0}$, the set $\Pi^{q}$ includes exotic non-equilibrium states with significant energy coherences such as the pure state $|\pi_{0}\rangle \langle \pi_{0}|$, where

(49) $$|\pi_{0}\rangle =\sum_{n}\sqrt{\frac{e^{-\beta \epsilon_{n}}}{Z^{q}}}|n\rangle .$$

Examples of this state arise in quantum optics [49,50].

More generally (that is, not restricting ourselves to the thermal energy equivalence class, Equation (48)), every quantum state $\hat{\rho }$ belongs to a unique energy equivalence class $\sum^{q}$ defined by the diagonal elements of $\hat{\rho }$ in the ${\hat{H}}_{0}$ basis. Within this class, the von Neumann entropy is maximized by the state $\hat{\sigma }\equiv \mathrm{diag}\;\hat{\rho }=\sum_{n}p_{n}|n\rangle \langle n|$:

(50a) $$\max_{\hat{\rho }\in \sum^{q}}S^{q}\left(\hat{\rho }\right)=S^{q}\left(\hat{\sigma }\right)=-\sum_{n}p_{n}\ln p_{n}\geq 0$$

where $p_{n}=\langle n|\hat{\rho }|n\rangle$. The von Neumann entropy is minimized within $\sum^{q}$ by pure states such as $|\psi \rangle \langle \psi |$, where $|\psi \rangle =\sum_{n}\sqrt{p_{n}}|n\rangle$, and for these states the entropy vanishes:

(50b) $$\min_{\hat{\rho }\in \sum^{q}}S^{q}\left(\hat{\rho }\right)=S^{q}\left(|\psi \rangle \langle \psi |\right)=0.$$

A classical energy equivalence class contains all phase space distributions ρ(Γ) with a given energy distribution η(E). An example is the thermal energy equivalence class

(51) $$\Pi^{c}=\left\{\rho \left(\Gamma \right)\;|\mathrm{diag}\;\rho \left(\Gamma \right)=\pi_{0}\left(\Gamma \right)\right\}$$

where $\pi_{0}\left(\Gamma \right)=\exp\left[-\beta H_{0}\left(\Gamma \right)\right]/Z^{c}$. While the state $\pi_{0}\left(\Gamma \right)$ is homogeneous, the class $\Pi^{c}$ contains states with substantial energy-shell inhomogeneities. For instance, if the system is a one-dimensional harmonic oscillator, the thermal equivalence class $\Pi^{c}$ includes the state

(52) $$\rho \left(E,T\right)=\underset{\eta_{\pi_{0}}\left(E\right)}{\underbrace{\left(\beta e^{-\beta E}\right)}}\underset{\zeta (T)}{\underbrace{\left(\omega \frac{e^{\delta \cos(\omega T)}}{2\pi I_{0}\left(\delta \right)}\right)}}$$

where *E* and *T* are the canonical energy and tempus (angle-like) coordinates [51] defined by $x=\sqrt{2E/m\omega^{2}}\cos\left(\omega T\right)$ and $p=\sqrt{2mE}\sin\left(\omega T\right)$, with ωT∈(−π,+π]; δ is a non-negative parameter; and $I_{0}$ is the modified Bessel function of order zero. For this example, it is convenient to use (E,T) rather than (x,p) to identify a point in classical phase space. ζ(T) is the von Mises distribution [52], an analogue of a Gaussian distribution for an angular coordinate. In Equation (52), the mean of ζ(T) is zero and its variance is controlled by δ. For δ=0, ρ(E,T) reduces to the canonical distribution, which is homogeneous over every energy shell. With increasing δ, the distribution becomes more and more concentrated on the positive *x*-axis of phase space (where T=0) and as a result its Shannon entropy $S^{c}\left[\rho \right]$ decreases, with no lower bound. Specifically, for large δ, we have

(53) $$S^{c}\left[\rho \left(E,T\right)\right]\approx -\ln\left(h\beta \omega \sqrt{\frac{\delta }{2\pi }}\right)+\frac{1}{2}\;,\;\delta \gg 1.$$

Every classical state ρ(Γ) belongs to a unique energy equivalence class $\sum^{c}$, defined by its energy distribution η(E) (Equation (37)). Within this class, the Shannon entropy is maximized by the diagonal state $\sigma \left(\Gamma \right)=\mathrm{diag}\;\rho \left(\Gamma \right)=\eta \left(H_{0}\right)/\Omega \left(H_{0}\right)$, but there is no lower bound on the minimum entropy, as the phase space distribution can be concentrated to an arbitrary degree without affecting the energy distribution:

(54) $$\begin{array}{cc} \max_{\rho \in \sum^{c}}S^{c}\left[\rho \left(\Gamma \right)\right] & =S^{c}\left[\sigma \left(\Gamma \right)\right]=-\int dE\;\eta \left(E\right)\ln\left[h^{N}\frac{\eta (E)}{\Omega (E)}\right] \end{array}$$

(55) $$\begin{array}{cc} \min_{\rho \in \sum^{c}}S^{c}\left[\rho \left(\Gamma \right)\right] & =-\infty . \end{array}$$

These extrema are illustrated by the values δ=0 and δ→∞ in the example in the previous paragraph.

**Figure 3:**
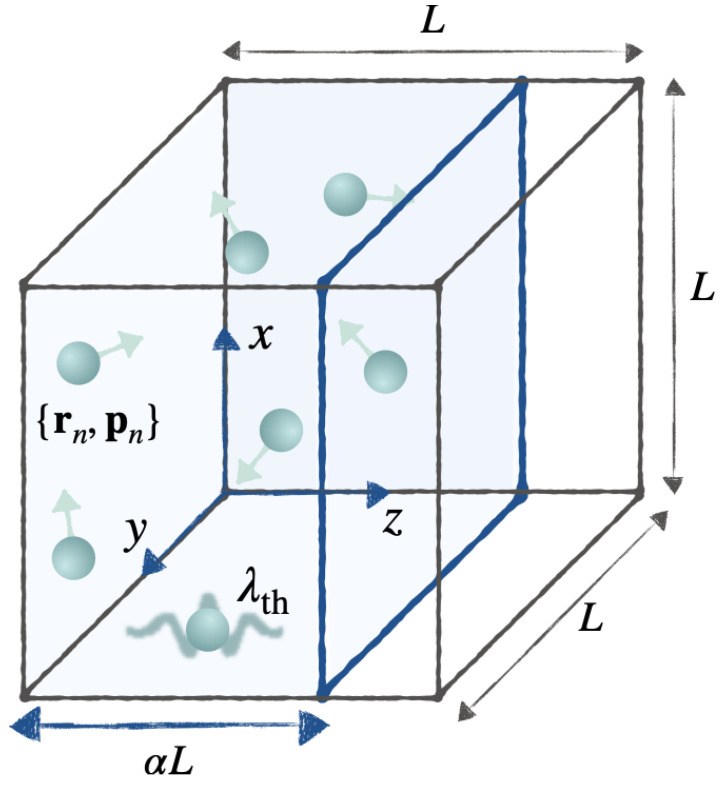
An ideal gas inside a box of volume $L^{3}$. The value of α∈(0,1] parametrizes a family of energy equivalence classes, with α=1 corresponding to thermal class $\Pi^{c}$. See text for details.

To take another illustrative example—which will prove useful in the next section—consider an ideal gas of *n* particles inside a three-dimensional cubic box of volume $V=L^{3}$, oriented parallel to the *x*-, *y*-, and *z*-axes, with one corner at the origin—see Figure 3. A point in phase space is given by $\Gamma =(r_{1}\cdots r_{n};p_{1}\cdots p_{n})$. For 0<α≤1, let $\rho_{\alpha }\left(\Gamma \right)$ denote the distribution for which the momenta $p_{k}$ are sampled from the Maxwellian distribution at temperature $\beta^{-1}$, and the positions $r_{k}$ are sampled uniformly within the region defined by 0<x,y<L and 0<z<αL. This distribution belongs to the thermal energy equivalence class $\Pi^{c}$, and $\rho_{\alpha =1}\left(\Gamma \right)$ is exactly the (homogeneous) thermal distribution, whereas $\rho_{\alpha <1}\left(\Gamma \right)$ is an inhomogeneous, non-equilibrium distribution, in which the gas is entirely located within a fraction α of the volume of the box. For arbitrary α∈(0,1], we have

(56) $$S^{c}\left[\rho_{\alpha }\left(\Gamma \right)\right]=n\ln\left(\frac{\alpha V}{\lambda_{\mathrm{th}}^{3}}\right)+\frac{3n}{2}$$

where $\lambda_{\mathrm{th}}=\sqrt{\beta h^{2}/2\pi m}$ is the thermal de Broglie wavelength. The value $S^{c}\left[\rho_{\alpha }\right]$ is maximized at α=1, that is, for the homogeneous state, and it has no lower bound as α→0.

In both of the above examples, by “squeezing” ρ(Γ) into an arbitrarily small region of phase space (δ→∞, α→0) we obtain a distribution with arbitrarily large, negative entropy.

### 4.2. An Unfair Comparison

We now determine the maximum amount of work that can be extracted in a cyclic isoenergetic process where all states in the quantum equivalence class $\sum^{q}$ are considered. Using Equations (21) and (50b), we have

(57) $$\begin{array}{cc} \max_{{\hat{\rho }}_{i}\in \sum^{q}}W^{q🟉}\left({\hat{\rho }}_{i}\right)\; & =\beta^{-1}\max_{{\hat{\rho }}_{i}\in \sum^{q}}\left[S^{q}\left(\mathrm{diag}\;{\hat{\rho }}_{i}\right)-S^{q}\left({\hat{\rho }}_{i}\right)\right]\\ \; & =\beta^{-1}\left[S^{q}\left(\hat{\sigma }\right)-\min_{{\hat{\rho }}_{i}\in \sum^{q}}S^{q}\left({\hat{\rho }}_{i}\right)\right]=\beta^{-1}S^{q}\left(\hat{\sigma }\right) \end{array}$$

where $\hat{\sigma }=\mathrm{diag}\;{\hat{\rho }}_{i}$ is the unique diagonal state belonging to $\sum^{q}$. The minimal value of $S^{q}\left({\hat{\rho }}_{i}\right)$ on the second line is achieved for any pure state $|\psi \rangle \langle \psi |\in \sum^{q}$, an example of which can always be constructed using the same argument as in Equation (50b). Hence, the maximum work is obtained by starting in a pure state, then quasistatically removing the coherences (e.g., following the protocol given by Equation (19)) so as to end in the diagonal state $\hat{\sigma }$. This result has a simple interpretation in terms of the bound $W^{q}\leq \beta^{-1}\left(S_{f}^{q}-S_{i}^{q}\right)$ (see Equation (16)): we maximize the extracted work by starting in a state with the lowest entropy and ending in the state of highest entropy, within $\sum^{q}$. By Equation (50) these are, respectively, any pure state and the unique diagonal state in $\sum^{q}$. Equivalently (since $U_{f}^{q}=U_{i}^{q}$ by Equation (13)), the maximum extracted work is obtained when starting in the state of highest free energy and ending in the state of lowest free energy. We emphasize that, here, free energy and entropy are defined by Equations (7) and (9), which apply to generic (not necessarily equilibrium) quantum states $\hat{\rho }$.

The analogous classical calculation, using Equations (47) and (55), gives

(58) $$\begin{array}{cc} \max_{\rho_{i}\in \sum^{c}}W^{c🟉}\left[\rho_{i}\left(\Gamma \right)\right]\; & =\beta^{-1}\max_{\rho_{i}\in \sum^{c}}\left(S^{c}\left[\mathrm{diag}\;\rho_{i}\left(\Gamma \right)\right]-S^{c}\left[\rho_{i}\left(\Gamma \right)\right]\right)\\ \; & =\beta^{-1}\left(S^{c}\left[\sigma \left(\Gamma \right)\right]-\min_{\rho_{i}\in \sum^{c}}S^{c}\left[\rho_{i}\left(\Gamma \right)\right]\right)=+\infty \end{array}$$

where $\sigma \left(\Gamma \right)=\mathrm{diag}\;\rho_{i}\left(\Gamma \right)$. In other words, for a given classical energy distribution, there is no upper bound on the amount of work that can be extracted, as there is no lower bound on the entropy of the initial state. By “squeezing” a given phase space distribution *within* each energy shell, without altering the distribution of probability *among* energy shells, we can construct a distribution $\rho_{i}\left(\Gamma \right)$ that is compressed within an arbitrarily small volume of phase space, hence we can make the value of $S^{c}\left[\rho_{i}\left(\Gamma \right)\right]$ arbitrarily small. This idea is illustrated by Equation (52) for the harmonic oscillator example of the previous section: as δ→∞, the von Mises distribution ζ(T) becomes ever more concentrated around T=0, and the entropy of the distribution becomes arbitrarily large and negative.

The example of the ideal gas discussed at the end of Section 4.1 provides further intuition for Equation (58). For that example, consider the thermal equivalence class $\Pi^{c}$, and imagine an initial inhomogeneous distribution $\rho_{i}\left(\Gamma \right)=\rho_{\alpha }\left(\Gamma \right)$ at t=0, with α<1, that is, with all gas particles initially located in the region 0<z<αL. To maximize the extracted work, we first suddenly insert a partition at the location z=αL, and then quasistatically move this partition to the location z=L, while the system remains in contact with a thermal bath at temperature $\beta^{-1}$. The process ends with the system in the homogeneous, thermal state $\rho_{f}\left(\Gamma \right)=\rho_{\alpha =1}\left(\Gamma \right)$. The total work extracted during this process of removing inhomogeneities is

(59) $$W^{c}=n\beta^{-1}\ln\frac{1}{\alpha }>0$$

which follows from a well-known expression for the reversible isothermal expansion of an ideal gas: $W=n\beta^{-1}\ln\left(V_{f}/V_{i}\right)$. It is easy to see why there is no upper bound on the extractable work: at $t=0^{+}$, just after the insertion of the partition, the gas is an equilibrium state, confined within a volume αV, with free energy $F^{c}\left(t=0^{+}\right)=-n\beta^{-1}\ln\left(\alpha V/\lambda_{\mathrm{th}}^{3}\right)$. The smaller the value of α, the larger the initial free energy and therefore the greater the amount of work that can be extracted through reversible, isothermal expansion. In this idealized example, we can begin with an arbitrarily dense initial state, i.e., arbitrarily small α>0.

In both the quantum and classical cases, the extracted work is maximized by evolving quasistatically from the state of lowest entropy to the state of highest entropy, within the equivalence class $\sum^{q}$ or $\sum^{c}$. Thus, there appears to be an inherent quantum thermodynamic *disadvantage*, since $S^{q}$ is bounded from below by 0, while $S^{c}$ is unbounded from below.

The comparison, however, is unfair. Quantum mechanics obeys the Heisenberg uncertainty principle, a loose semiclassical interpretation of which states that every quantum state occupies a cell of volume $h^{N}$ in phase space. If we view classical mechanics as an approximate model of an underlying quantum reality, then when considering initial distributions $\rho_{i}\left(\Gamma \right)$ we should allow only such distributions as are consistent with the uncertainty principle. To impose this constraint, let us imagine dividing phase space into cells of volume $h^{N}$. A distribution ρ(Γ) that is consistent with the uncertainty principle is one that is uniform within any such cell, but whose value differs from cell to cell: any finer-grained structure is offensive to the uncertainty principle. For such a distribution, we have $p_{k}=h^{N}\rho \left(\Gamma_{k}\right)$, where $\Gamma_{k}$ is a representative point in cell *k* and $p_{k}=\int_{\Gamma \in \mathrm{cell}\;k}d\Gamma \;\rho \left(\Gamma \right)$ is the probability to find the system in that cell. The Shannon entropy of this distribution is given by

(60) $$S^{c}\left[\rho \right]=-\int \rho \left(\Gamma \right)\ln\left[h^{N}\rho \left(\Gamma \right)\right]d\Gamma =-\sum_{k}p_{k}\ln p_{k}\geq 0$$

where $S^{c}=0$ if and only if $p_{k}=\delta_{kl}$ for some cell *l*.

If we thus reject distributions with negative entropy as being incompatible with the uncertainty principle, then Equation (55) is replaced by $\min_{\rho \in \sum^{c}}S^{c}\left[\rho \left(\Gamma \right)\right]=0$, and Equation (58) becomes

(61) $$\max_{\rho_{i}\in \sum^{c}}W^{c🟉}\left[\rho_{i}\left(\Gamma \right)\right]=\beta^{-1}S^{c}\left[\sigma \left(\Gamma \right)\right]\;.$$

Thus, after imposing consistency with the uncertainty principle (in an admittedly heuristic fashion), we conclude that for both the quantum equivalence class $\sum^{q}$ and the classical equivalence class $\sum^{c}$, the maximum extractable work is given by the entropy of the diagonal or homogeneous state, multiplied by $\beta^{-1}$ (Equations (57) and (61)).

Throughout the following section, and in Section 5, we impose the constraint $S^{c}\left[\rho \right]\geq 0$ on the initial classical phase space distribution, to exclude states that are incompatible with the uncertainty principle.

### 4.3. A Fair Comparison

The final step in making a fair comparison between quantum and classical work extraction is to establish a correspondence between equivalence classes $\sum^{q}$ and $\sum^{c}$. That is, we want to establish a correspondence between quantum and classical energy distributions. There is no unique way to do this, as energy takes on discrete values in one case and continuous values in the other. As a reasonable way to proceed, let us choose a real function κ(·)≥0 with the property that both $K^{q}=\mathrm{Tr}\;\kappa \left({\hat{H}}_{0}\right)$ and $K^{c}=\int d\Gamma \;\kappa \left(H_{0}\left(\Gamma \right)\right)$ are finite. We then define the diagonal quantum and homogeneous classical states

(62) $${\hat{\sigma }}_{\kappa }=\frac{\kappa ({\hat{H}}_{0})}{K^{q}}=\sum_{n}\frac{\kappa (\epsilon_{n})}{K^{q}}|n\rangle \langle n|\;,\;\sigma_{\kappa }\left(\Gamma \right)=\frac{\kappa (H_{0}\left(\Gamma \right))}{K^{c}}$$

along with the associated energy equivalence classes

(63a) $$\begin{array}{ccc} \sum^{q}\left[\kappa \right] & = & \left\{\hat{\rho }\;|\mathrm{diag}\;\hat{\rho }={\hat{\sigma }}_{\kappa }\right\} \end{array}$$

(63b) $$\begin{array}{ccc} \sum^{c}\left[\kappa \right] & = & \{\rho \left(\Gamma \right)\;|\mathrm{diag}\;\rho =\sigma_{\kappa }\;,\;S^{c}\left[\rho \right]\geq 0\}. \end{array}$$

The equivalence class $\sum^{q}\left[\kappa \right]$ contains all quantum states with diagonal density matrix elements $\rho_{nn}=\kappa \left(\epsilon_{n}\right)/K^{q}$, whereas $\sum^{c}\left[\kappa \right]$ contains every classical state with energy distribution

(64) $$\eta \left(E\right)=\frac{\kappa (E)\Omega (E)}{K^{c}}.$$

Thus, a given choice of κ(·) specifies both a quantum and a classical energy distribution. As an example, for the choice $\kappa \left(x\right)=e^{-\beta x}$, the reference states are ${\hat{\sigma }}_{\kappa }={\hat{\pi }}_{0}$ and $\sigma_{\kappa }\left(\Gamma \right)=\pi_{0}\left(\Gamma \right)$, and the energy equivalence classes are the thermal sets defined earlier: $\sum^{q}\left[\kappa \right]=\Pi^{q}$ and $\sum^{c}\left[\kappa \right]=\Pi^{c}$.

In the semiclassical limit h→0, as the level spacing between adjacent energy eigenvalues approaches zero, the normalized energy distribution associated with $\sum^{q}\left[\kappa \right]$ is conveniently written as $\xi \left(E\right)=\kappa \left(E\right)g\left(E\right)/K^{q}$, where $g\left(E\right)=\sum_{n}\delta \left(E-\epsilon_{n}\right)$ is the quantum density of states. In turn, g(E)dE is approximated by the number of cells of volume $h^{N}$ that fit into the classical phase space volume between *E* and E+dE, for small dE. Equivalently,

(65) $$\lim_{h\to 0}h^{N}g\left(E\right)=\Omega \left(E\right)$$

where Ω(E) is the classical density of states, Equation (39). Hence, the quantum energy distribution is, semiclassically,

(66) $$\xi \left(E\right)=\frac{\kappa (E)\Omega (E)}{h^{N}K^{q}}.$$

Since both the classical and quantum energy distributions η(E) and ξ(E) (Equations (64) and (66)) are normalized to unity, we have

(67) $$\lim_{h\to 0}h^{N}K^{q}=K^{c}.\;$$

From Equations (64), (66) and (67), we conclude that in the semiclassical limit h→0, the discrete energy distribution associated with the equivalence class $\sum^{q}\left[\kappa \right]$ approaches the continuous distribution associated with $\sum^{c}\left[\kappa \right]$. In this sense, we view $\sum^{q}\left[\kappa \right]$ and $\sum^{c}\left[\kappa \right]$ as having equivalent energy distributions.

Now, finally, for a given quantum reference Hamiltonian ${\hat{H}}_{0}$ and its classical counterpart $H_{0}\left(\Gamma \right)$, and for a given choice of the function κ(·), let

(68) $$W_{\max}^{q🟉}\left[\kappa \right]=\max_{{\hat{\rho }}_{i}\in \sum^{q}\left[\kappa \right]}W^{q🟉}\left({\hat{\rho }}_{i}\right)\;\mathrm{and}\;W_{\max}^{c🟉}\left[\kappa \right]=\max_{\rho_{i}\in \sum^{c}\left[\kappa \right]}W^{c🟉}\left[\rho_{i}\left(\Gamma \right)\right]$$

denote the maximum quantum and classical work that can be extracted during a cyclic, isoenergetic (in the sense of Equations (13) and (42)) process, for initial energy distributions determined by κ(·). We assert that by comparing the values of $W_{\max}^{q🟉}\left[\kappa \right]$ and $W_{\max}^{c🟉}\left[\kappa \right]$, in the semiclassical limit h→0, we make a fair comparison between quantum work that can be extracted from coherences, and classical work that can be extracted from inhomogeneities.

From Equations (57), (61) and (63), we have

(69) $$W_{\max}^{q🟉}\left[\kappa \right]=\beta^{-1}S^{q}\left({\hat{\sigma }}_{\kappa }\right)\;,\;W_{\max}^{c🟉}\left[\kappa \right]=\beta^{-1}S^{c}\left[\sigma_{\kappa }\left(\Gamma \right)\right],$$

therefore, let us inspect the difference between these two values,

(70) $$\Delta W^{🟉}=W_{\max}^{q🟉}\left[\kappa \right]-W_{\max}^{c🟉}\left[\kappa \right],\;$$

in the limit h→0. Following the semiclassical approach used above, we obtain

(71) $$\begin{array}{cc} \lim_{h\to 0}\Delta W^{🟉}\; & =\beta^{-1}\lim_{h\to 0}\left\{-\sum_{n}\frac{\kappa (\epsilon_{n})}{K^{q}}\ln\left[\frac{\kappa (\epsilon_{n})}{K^{q}}\right]+\int \frac{\kappa (H_{0})}{K^{c}}\ln\left[h^{N}\frac{\kappa (H_{0})}{K^{c}}\right]d\Gamma \right\}\\ \; & =\beta^{-1}\lim_{h\to 0}\left\{-\int g\left(E\right)\frac{\kappa (E)}{K^{q}}\ln\left[\frac{\kappa (E)}{K^{q}}\right]dE+\int \Omega \left(E\right)\frac{\kappa (E)}{K^{c}}\ln\left[h^{N}\frac{\kappa (E)}{K^{c}}\right]dE\right\}\\ \; & =\beta^{-1}\lim_{h\to 0}\left\{-\int \Omega \left(E\right)\frac{\kappa (E)}{K^{c}}\ln\left[h^{N}\frac{\kappa (E)}{K^{c}}\right]dE+\int \Omega \left(E\right)\frac{\kappa (E)}{K^{c}}\ln\left[h^{N}\frac{\kappa (E)}{K^{c}}\right]dE\right\}\\ \; & =0. \end{array}$$

Here, Equation (62) has been combined with the expressions for von Neumann and Shannon entropy (Equations (9) and (31)) on the first line; the sum over energy eigenstates and the integral over phase space have been replaced by energy integrals on the second line; and Equations (65) and (67) have been used to get to the third line.

For $\kappa \left(x\right)=e^{-\beta x}$, Equation (71) can alternatively be established from the result (see Equations (4) and (26))

(72) $$\Delta W^{🟉}=\beta^{-1}\left\{S^{q}\left({\hat{\pi }}_{0}\right)-S^{c}\left[\pi_{0}\left(\Gamma \right)\right]\right\}=\beta \frac{\partial }{\partial \beta }\left(F^{q,\mathrm{eq}}-F^{c,\mathrm{eq}}\right)=\left(-\frac{1}{\beta }+\frac{\partial }{\partial \beta }\right)\log\frac{Z^{c}}{h^{N}Z^{q}}$$

where $Z^{q}$ and $Z^{c}$ are equilibrium partition functions. Taking the limit h→0 and using the known result [47,53,54,55] that (for kinetic-plus-potential Hamiltonians) $h^{N}Z^{q}$ can be expanded in a power series of *h* whose first term is exactly the classical partition function $Z^{c}$, the right side of Equation (72) vanishes.

From Equation (71), we conclude that in the semiclassical limit, the maximal work that can be extracted from the energy coherences of a quantum state ${\hat{\rho }}_{i}\in \sum^{q}\left[\kappa \right]$ is the same as the maximal work that can be extracted from the energy-shell inhomogeneities of a classical state $\rho_{i}\left(\Gamma \right)\in \sum^{c}\left[\kappa \right]$. In both situations, the work is maximized by starting in the state of least entropy within $\sum^{q}$ or $\sum^{c}$, then quasistatically removing the coherences or inhomogeneities. This result leads us to conclude that, within our framework for comparing quantum and classical systems, quantum coherences offer no particular thermodynamic advantage over classical inhomogeneities.

## 5. Dropping the Isoenergetic Constraint

In the previous sections, we have imposed the isoenergetic constraint, namely that the initial and final energy distributions are identical (Equations (13) and (42)). Let us now drop this constraint and pose the following question. For a quantum or classical system described by an initial Hamiltonian ${\hat{H}}_{0}$ or $H_{0}\left(\Gamma \right)$, in the presence of a thermal bath at temperature $\beta^{-1}$, what is the maximum work that can be extracted during a cyclic process if the energy distribution of the initial state is determined by a given function κ(·)?

In the quantum case, we first let $W^{q†}\left({\hat{\rho }}_{i}\right)$ denote the maximum work extracted for a given initial state ${\hat{\rho }}_{i}$—this quantity is analogous to $W^{🟉}\left({\hat{\rho }}_{i}\right)$ (Section 2) but without the constraint $\mathrm{diag}\;{\hat{\rho }}_{f}=\mathrm{diag}\;{\hat{\rho }}_{i}$. From Equations (5) and (10) and the non-negativity of the Kullback–Leibler divergence, we have

(73) $$\begin{array}{cc} W^{q†}\left({\hat{\rho }}_{i}\right) & \leq F^{q}\left({\hat{\rho }}_{i},{\hat{H}}_{0}\right)-F^{q}\left({\hat{\rho }}_{f},{\hat{H}}_{0}\right)\\ \; & \leq F^{q}\left({\hat{\rho }}_{i},{\hat{H}}_{0}\right)-F^{q}\left({\hat{\pi }}_{0},{\hat{H}}_{0}\right)\\ \; & =U_{i}^{q}-\beta^{-1}S_{i}^{q}-F_{0}^{q,\mathrm{eq}} \end{array}$$

where the inequality on the first line is valid for any final state ${\hat{\rho }}_{f}$, and $F_{0}^{q,\mathrm{eq}}\equiv F^{q,\mathrm{eq}}\left({\hat{H}}_{0}\right)$. As shown in Appendix A, the bound obtained in Equation (73) is saturated by the protocol

(74) $$\hat{H}\left(t\right)=\left\{\begin{array}{cc} {\hat{H}}_{0} & \;t\leq 0\\ -\beta^{-1}\ln\left[\left(1-\lambda \right){\hat{\rho }}_{i}+\lambda \;e^{-\beta {\hat{H}}_{0}}\right] & \;0<t\leq \tau \\ {\hat{H}}_{0} & \;\tau \leq t \end{array}\right.$$

where λ≡t/τ and the process is quasistatic: τ→∞. (Note that there is no quench at t=τ.) Since the bound can be saturated, and $W^{q†}\left({\hat{\rho }}_{i}\right)$ was defined as the maximum work that can be extracted, we simply write

(75) $$W^{q†}\left({\hat{\rho }}_{i}\right)=U_{i}^{q}-\beta^{-1}S_{i}^{q}-F_{0}^{q,\mathrm{eq}}.$$

Now, maximizing this quantity over all ${\hat{\rho }}_{i}\in \sum^{q}\left[\kappa \right]$, we have

(76) $$\begin{array}{cc} W_{\max}^{q†}\left[\kappa \right] & =\max_{{\hat{\rho }}_{i}\in \sum^{q}\left[\kappa \right]}\left(U_{i}^{q}-\beta^{-1}S_{i}^{q}-F_{0}^{q,\mathrm{eq}}\right)\\ \; & =\max_{{\hat{\rho }}_{i}\in \sum^{q}\left[\kappa \right]}\left(U^{q}\left[\kappa \right]-\beta^{-1}S_{i}^{q}-F_{0}^{q,\mathrm{eq}}\right)\\ \; & =U^{q}\left[\kappa \right]-F_{0}^{q,\mathrm{eq}} \end{array}$$

where $U^{q}\left[\kappa \right]\equiv \left(1/K^{q}\right)\sum_{n}\kappa \left(\epsilon_{n}\right)\epsilon_{n}$ is the average energy for every state ${\hat{\rho }}_{i}\in \sum^{q}\left[\kappa \right]$, and we have used Equation (50b) to arrive at the third line.

As a consistency check, we combine Equations (69) and (76) with Equations (7) and (10) to obtain

(77) $$\begin{array}{cc} W_{\max}^{q†}\left[\kappa \right]-W_{\max}^{q🟉}\left[\kappa \right]\; & =U^{q}\left[\kappa \right]-F_{0}^{q,\mathrm{eq}}-\beta^{-1}S^{q}\left({\hat{\sigma }}_{\kappa }\right)\\ \; & =F^{q}\left({\hat{\sigma }}_{\kappa },{\hat{H}}_{0}\right)-F_{0}^{q,\mathrm{eq}}=D\left({\hat{\sigma }}_{\kappa }|{\hat{\pi }}_{0}\right)\geq 0 \end{array}$$

where ${\hat{\sigma }}_{\kappa }$ is the unique diagonal state belonging to $\sum^{q}\left[\kappa \right]$. Thus, $W_{\max}^{q†}\left[\kappa \right]\geq W_{\max}^{q🟉}\left[\kappa \right]$, which makes sense: the maximum work that we can extract without imposing the constraint $\mathrm{diag}\;{\hat{\rho }}_{f}=\mathrm{diag}\;{\hat{\rho }}_{i}$ must be no less than the maximum work we can extract with the constraint.

In the classical case, essentially identical calculations—which we do not reproduce here—lead to the result

(78) $$W_{\max}^{c†}\left[\kappa \right]=U^{c}\left[\kappa \right]-F_{0}^{c,\mathrm{eq}}$$

where $W_{\max}^{c†}\left[\kappa \right]$ is the maximum work that can be extracted over all initial states $\rho_{i}\left(\Gamma \right)\in \sum^{c}\left[\kappa \right]$, without imposing Equation (42), and $U^{c}\left[\kappa \right]=\left(1/K^{c}\right)\int d\Gamma \;\kappa \left(H_{0}\right)H_{0}$ is the average energy for every state in $\sum^{c}\left[\kappa \right]$. Following steps similar to those of Section 4.3, we obtain

(79) $$\begin{array}{cc} \lim_{h\to 0}U^{q}\left[\kappa \right] & =\lim_{h\to 0}\frac{1}{K^{q}}\sum_{n}\kappa \left(\epsilon_{n}\right)\epsilon_{n}\\ \; & =\lim_{h\to 0}\int dE\;g\left(E\right)\frac{\kappa (E)}{K^{q}}E\\ \; & =\int dE\;\Omega \left(E\right)\frac{\kappa (E)}{K^{c}}E=\frac{1}{K^{c}}\int d\Gamma \;\kappa \left(H_{0}\right)\;H_{0}=U^{c}\left[\kappa \right] \end{array}$$

and

(80) $$\lim_{h\to 0}\left(F_{0}^{c,\mathrm{eq}}-F_{0}^{q,\mathrm{eq}}\right)=-\beta^{-1}\lim_{h\to 0}\ln\frac{Z^{c}\left[H_{0}\right]}{h^{N}Z^{q}\left({\hat{H}}_{0}\right)}=0$$

using Equation (67), with $\kappa \left(x\right)=e^{-\beta x}$.

Defining $\Delta W^{†}\equiv W_{\max}^{q†}\left[\kappa \right]-W_{\max}^{c†}\left[\kappa \right]$, Equations (76) and (78)–(80) give us

(81) $$\lim_{h\to 0}\Delta W^{†}=0$$

which is the counterpart of Equation (71), after abandoning the constraint of equal initial and final energy distributions. We again conclude that quantum coherences provide no inherent thermodynamic advantage over classical inhomogeneities, in the semiclassical limit.

## 6. Conclusions

In Section 2 and Section 3 of this paper, we argued that quantum energy coherences (as shown earlier [9]) and classical energy shell inhomogeneities represent thermodynamic resources, which can be leveraged to deliver work. In Section 4 and Section 5, we argued that a fair comparison shows these resources to be equivalent: in the semiclassical limit, and for a given initial energy distribution, the amount of work that can be extracted from quantum coherences is the same as the amount that can be extracted from classical inhomogeneities.

Our study has focused on processes during which the system of interest is in contact with a thermal reservoir, and here (as we have seen) the free energy F plays an important role. Sone and Deffner [18] have recently carried out a similar investigation for isolated quantum and classical systems, in which case *ergotropy* (defined in Ref. [1] for quantum systems and in Ref. [18] for classical systems) plays a role analogous to free energy in our paper. In Ref. [18], as in our paper, energy-shell inhomogeneities are classical counterparts of quantum energy coherences.

In making our comparison in Section 4 and Section 5, we invoked a quantum–classical correspondence based on canonical quantization, in which the system of interest is described by coordinates $x_{1},\;x_{2},\cdots$ and conjugate momenta $p_{1},\;p_{2},\cdots$, which are either quantum operators or classical observables. For such systems, the classical phase space is unbounded and the quantum Hilbert space is infinite-dimensional.

However, in the quantum thermodynamics literature one often encounters systems with finite-dimensional Hilbert spaces, such as the illustrative qubit example analyzed in Ref. [9]. It then seems natural to take, as the quantum system’s counterpart, a discrete-state classical system of equal dimensionality. Thus, a qubit’s counterpart may be taken to be a classical bit. For such discrete-state systems there is no opportunity to introduce a classical analogue of quantum coherences, as the statistical state of a classical *D*-state system is specified *entirely* by the probabilities $P_{1},\cdots P_{D}$, and these are in one-to-one correspondence with the diagonal elements of the corresponding quantum system’s density matrix $\hat{\rho }$. In this situation, it seems that quantum coherences really do provide a unique thermodynamic resource that is unavailable to classical counterparts.

This conclusion, however, is misleading, as an apparently discrete-state classical system is in reality a coarse-grained version of a more microscopically detailed system. For example, an effective classical bit can be obtained by coarse-graining a classical particle in a double-well potential, such that the location *x* of the particle in the left (right) well indicates a bit value of 0 (1). The apparent quantum thermodynamic advantage—due to coherences—arises in this case because potentially useful classical information (e.g., how the particle’s potential energy depends on its location *x*) has been thrown out in the process of coarse-graining from the double well to the bit. Comparing a qubit—an intrinsically two-state quantum system—with an effective classical two-state system obtained by discarding microscopic information, is an apples-to-oranges comparison.

There is no generally applicable procedure for identifying a proper classical counterpart of a quantum system with a finite-dimensional Hilbert space. It is instructive, however, to consider the simplest case of a spin-1/2 particle (qubit) in a magnetic field, governed by a Hamiltonian $\hat{H}=g\;B\cdot \hat{s}$, where $\hat{s}=\left(\hbar /2\right)\left({\hat{\sigma }}_{x},{\hat{\sigma }}_{y},{\hat{\sigma }}_{z}\right)$. In the absence of a thermal bath, the unitary dynamics in the Heisenberg representation are given by the equations of motion

(82) $$i\hbar \frac{d}{dt}{\hat{s}}_{H}=\left[{\hat{s}}_{H},\hat{H}\right]$$

where the right side is evaluated using the commutation relations

(83) $$\left[{\hat{s}}_{j},{\hat{s}}_{k}\right]=i\hbar \;\varepsilon_{jkl}\;{\hat{s}}_{l}$$

and $\varepsilon_{jkl}$ is the Levi-Civita symbol. Kammerlander and Anders [9] showed how work can be extracted from energy coherences in such a system, using a protocol involving quenches and the quasistatic variation of B, along with coupling to a thermal bath.

As a possible classical counterpart, instead of a two-state bit let us consider a system whose microscopic state is described by a vector $S=(S_{x},S_{y},S_{z})$ of fixed magnitude, governed by a Hamiltonian H=gB·S, evolving under the Poisson bracket formulation of Hamiltonian dynamics,

(84) $$\frac{d}{dt}S=\left\{S,H\right\}$$

with

(85) $$\left\{S_{j},S_{k}\right\}=\varepsilon_{jkl}\;S_{l}.$$

The phase space for this classical system is bounded: it is the two-dimensional surface of a sphere of radius |S|. An energy shell is represented by a circle on that sphere, oriented along the B-direction. The dynamics given by Equation (84) describe an isolated system, and would have to be supplemented by appropriate terms in order to include the effects of contact with a thermal bath. It would then be interesting to investigate classical protocols designed to extract work from an initial distribution that is inhomogeneous on the energy shells, and to compare this classical situation with the quantum case of Ref. [9].

We note that the approach described in the previous paragraphs is readily extended to a system composed of N>1 spins, interacting both with external fields and among themselves, e.g., through Hamiltonian terms of the form $c_{mn}{\hat{s}}_{m}\cdot {\hat{s}}_{n}$ or $c_{mn}S_{m}\cdot S_{n}$. Thus, comparisons between quantum and classical work extraction can be extended to multi-spin systems, within this framework. For example, it has been demonstrated that quantum correlations within a many-body system can be utilized for extracting work [56,57,58,59], and it would be pertinent to study whether one can leverage classical correlations and inhomogeneities in a similar way. Such comparisons may further elucidate whether thermodynamic advantages can be identified that are unique to quantum systems.

## Appendix A

Here, we show that the work bounds appearing in Equations (18), (45) and (73) are saturated by the protocols given by Equations (19), (46) and (74), when these protocols are performed quasistatically (τ→∞). In both the quantum and classical cases, our key assumption is that in the absence of driving the system relaxes to the thermal equilibrium state. Hence, under quasistatic driving, the system tracks the instantaneous equilibrium state.

In the quantum case, we first note that for a time-dependent Hamiltonian $\hat{H}\left(t\right)$ and the corresponding equilibrium state $\hat{\pi }\left(t\right)$ and free energy $F^{q,\mathrm{eq}}\left(t\right)=-\beta^{-1}\ln Z^{q}\left(t\right)$, the following relation holds:

(A1) $$\frac{d}{dt}F^{q,\mathrm{eq}}\left(t\right)=\mathrm{Tr}\left[\frac{d\hat{H}}{dt}\hat{\pi }\right]$$

as can be verified directly from Equations (3) and (4). This identity is the quantum counterpart of the classical thermodynamic integration identity, Equation (A8), which underlies the thermodynamic integration technique for free energy estimation [60,61]. Equation (A1) states that the rate of change in the equilibrium free energy, with respect to time, is the equilibrium average of the rate of change in the Hamiltonian.

Now, consider a quantum system whose initial state is ${\hat{\rho }}_{i}$, which is driven according to the protocol given by Equation (19). The initial step of this protocol is an instantaneous change in the Hamiltonian from $\hat{H}\left(0\right)={\hat{H}}_{0}$ to $\hat{H}\left(0^{+}\right)=-\beta^{-1}\ln{\hat{\rho }}_{i}$. The state of the system does not change during this step. The resulting work performed is

(A2) $$\begin{array}{cc} W_{0\to 0^{+}}^{q}\; & =-\int_{0}^{0^{+}}\mathrm{Tr}\left[\frac{d\hat{H}}{dt}\hat{\rho }\right]dt\\ \; & =\mathrm{Tr}\left[\left(\hat{H}\left(0\right)-\hat{H}\left(0^{+}\right)\right){\hat{\rho }}_{i}\right]\\ \; & =\mathrm{Tr}\left[\left({\hat{H}}_{0}+\beta^{-1}\ln{\hat{\rho }}_{i}\right){\hat{\rho }}_{i}\right]\\ \; & =U_{i}^{q}-\beta^{-1}S_{i}^{q}=F_{i}^{q}. \end{array}$$

During the quasistatic stage of the protocol, the system evolves through a sequence of equilibrium states, $\hat{\rho }\left(t\right)=\hat{\pi }\left(t\right)$—see Equation (20). Applying Equation (A1), the resulting work is

(A3) $$\begin{array}{cc} W_{0^{+}\to \tau^{-}}^{q}\; & =-\int_{0^{+}}^{\tau^{-}}\mathrm{Tr}\left[\frac{d\hat{H}}{dt}\hat{\pi }\right]\;dt\\ \; & =-\int_{0^{+}}^{\tau^{-}}\frac{d}{dt}F^{q,\mathrm{eq}}\left(t\right)\;dt\\ \; & =F^{q,\mathrm{eq}}\left(\tau^{-}\right)-F^{q,\mathrm{eq}}\left(0^{+}\right)\\ \; & =-\beta^{-1}\ln\mathrm{Tr}\;e^{-\beta \hat{H}\left(\tau^{-}\right)}+\beta^{-1}\ln\mathrm{Tr}\;e^{-\beta \hat{H}\left(0^{+}\right)}\\ \; & =-\beta^{-1}\ln\frac{\mathrm{Tr}\;\mathrm{diag}\;{\hat{\rho }}_{i}}{\mathrm{Tr}\;{\hat{\rho }}_{i}}=0. \end{array}$$

Proceeding as in Equation (A2), we obtain

(A4) $$W_{\tau^{-}\to \tau }^{q}=-F_{f}^{q}$$

for the Hamiltonian quench at t=τ. Thus, the total work over the entire process is

(A5) $$W^{q}=F_{i}^{q}-F_{f}^{q}=\beta^{-1}\left[S^{q}\left(\mathrm{diag}\;{\hat{\rho }}_{i}\right)-S^{q}\left({\hat{\rho }}_{i}\right)\right]$$

(using $U_{i}^{q}=U_{f}^{q}$), as claimed at the end of Section 2.

Similar calculations for the protocol appearing in Equation (74) give

(A6a) $$\begin{array}{cc} W_{0\to 0^{+}}^{q} & =U_{i}^{q}-\beta^{-1}S_{i}^{q} \end{array}$$

(A6b) $$\begin{array}{cc} W_{0^{+}\to \tau^{-}}^{q} & =-F_{0}^{q,\mathrm{eq}} \end{array}$$

(A6c) $$\begin{array}{cc} W_{\tau^{-}\to \tau }^{q} & =0, \end{array}$$

hence,

(A7) $$W^{q}=U_{i}^{q}-\beta^{-1}S_{i}^{q}-F_{0}^{q,\mathrm{eq}}$$

as claimed in Section 5, just after Equation (74).

In the classical case, for a Hamiltonian H(Γ,t) we have the identity

(A8) $$\frac{d}{dt}F^{c,\mathrm{eq}}\left(t\right)=\int d\Gamma \;\frac{\partial H}{\partial t}\left(\Gamma ,t\right)\pi \left(\Gamma ,t\right).$$

For the protocol given by Equation (46), calculations essentially identical to those appearing above in Equations (A2)–(A4), but with quantum traces replaced by integrals over classical phase space, give

(A9a) $$\begin{array}{cc} W_{0\to 0^{+}}^{c} & =F_{i}^{c} \end{array}$$

(A9b) $$\begin{array}{cc} W_{0^{+}\to \tau^{-}}^{c} & =0 \end{array}$$

(A9c) $$\begin{array}{cc} W_{\tau_{-}\to \tau_{+}}^{c} & =-F_{f}^{c}, \end{array}$$

hence,

(A10) $$W^{c}=F_{i}^{c}-F_{f}^{c}=\beta^{-1}\left(S^{c}\left[\mathrm{diag}\;\rho_{i}\right]-S^{c}\left[\rho_{i}\right]\right)$$

as claimed at the end of Section 3.2.
